# Police Training in Practice: Organization and Delivery According to European Law Enforcement Agencies

**DOI:** 10.3389/fpsyg.2021.798067

**Published:** 2022-01-17

**Authors:** Lisanne Kleygrewe, Raôul R. D. Oudejans, Matthijs Koedijk, R. I. (Vana) Hutter

**Affiliations:** ^1^Department of Human Movement Sciences, Amsterdam Movement Sciences, Vrije Universiteit Amsterdam, Amsterdam, Netherlands; ^2^Institute of Brain and Behaviour Amsterdam, Amsterdam, Netherlands; ^3^Faculty of Sports and Nutrition, Amsterdam University of Applied Sciences, Amsterdam, Netherlands; ^4^Netherlands Institute for the Study of Crime and Law Enforcement, Nederlands Studiecentrum Criminaliteit en Rechtshandhaving, Amsterdam, Netherlands

**Keywords:** police training, didactics, curriculum evaluation, police instructors, assessment

## Abstract

Police training plays a crucial role in the development of police officers. Because the training of police officers combines various educational components and is governed by organizational guidelines, police training is a complex, multifaceted topic. The current study investigates training at six European law enforcement agencies and aims to identify strengths and challenges of current training organization and practice. We interviewed a total of 16 police instructors and seven police coordinators with conceptual training tasks. A thematic analysis (Braun and Clarke, 2006; Terry et al., 2017) was conducted and results organized in the two main themes evident across all six law enforcement agencies: organization of training and delivery of training. Results show that governmental structures and police executive boards are seen as the primary authorities that define the training framework in which police instructors operate. These administrative structures regulate distant and immediate resources, such as available training time, training facilities, equipment, and personnel. Within the confines of available resources and predetermined training frameworks, results indicate that police instructors thoroughly enjoy teaching, creating supportive and motivating learning environments, and applying their personal learning perspectives to training. Nonetheless, police instructors are critical of the level of training they are able to achieve with the available resources.

## Introduction

Police training plays a crucial role in the development of police officers. Compared to other occupations, police officers spend the entire beginning of their policing career training and preparing for the job (Wilson et al., 2010). Police cadets may spend up to three years in basic training before they are considered police officers and encounter any job-specific situations independently. This comparatively long period of initial training makes sense when considering that police officers respond to diverse and complex on-duty demands on a daily basis (Anderson et al., 2002; Gershon et al., 2009; Paton, 2009). Police officers are tasked with enforcing laws, protecting civilian life and property, responding to (emergency) calls, and apprehending and arresting criminals, to name only a few. Consequently, it is likely for police officers to encounter complex, high-risk situations (Marenin, 2004; Waddington et al., 2012). Dealing with these high-risk situations adequately requires expansive knowledge and skills, which police officers ought to acquire in training. Police academies and law enforcement agencies are responsible for equipping officers with the relevant skills to successfully resolve any on-duty demands placed upon them (Chappell, 2008).

The common objective of police training has hardly changed over time – to help police officers perform their job (Ness, 1991; Koedijk et al., 2019). However, what police training consists of has changed significantly over the years. Traditional policing required police officers to possess self-defense, arresting, shooting, and driving skills, which was reflected in training that focused primarily on teaching these physical activities (Chappell, 2008). Current policing places a focus on additional skills such as communication, problem-solving, and decision-making (Birzer and Tannehill, 2001; Blumberg et al., 2019). To facilitate these skills in the context of policing, police academies and law enforcement agencies needed to adjust the structure, content, and delivery of their formal training (Marenin, 2004). Traditionally, police instructors taught their students knowledge and skills using a uniform, linear training approach (Birzer and Tannehill, 2001; McCoy, 2006). For instance, teaching cadets self-defense skills would require instructors to explain the exact techniques and to illustrate a fixed set of movements for cadets to observe and apply in a static, low pressure setting. Considering that police officers encounter complex and dynamic incidents, where decision-making, situational awareness, and communication skills might be decisive for the outcome, the traditional, uniform approach to training seems to have little to do with the realities of police work (Renden et al., 2015).

Recent literature in the field of police training investigated how to better facilitate skills and improve specific components of police training. For instance, Di Nota and Huhta (2019) have illustrated how realistic and immersive scenario-based training can improve police officers’ skills such as situational awareness and decision-making. Similarly, integrating elements of anxiety and stress into training — akin to what police officers would experience in high-risk on-duty situations — has shown to improve use of force performance under stressful conditions and paved the path for police training to become more realistic (Oudejans, 2008; Nieuwenhuys and Oudejans, 2011; Andersen et al., 2016). Furthermore, police instructors are called to create learner-centered training environments that foster the “exploration and learning of functional solutions” to reduce the gap between police training and police work on duty (Koerner and Staller, 2021, p. 10; White and Escobar, 2008). This means moving away from traditional classroom-based, trainer-centered teaching to enhance performance, skill transfer, and retention.

While research has contributed immensely to the quality of police training, current literature is yet to provide a comprehensive overview of police training across law enforcement agencies. Two reasons, in particular, might explain the lack of a cross-cultural overview of police training. First, almost every law enforcement agency organizes the frequency, duration, and content of their training differently (Marenin, 2004). This is due to many factors. The availability of resources and budget for training may determine how much and how often training can be conducted (White and Escobar, 2008), while the content of training will vary as it is tailored to particular needs of the region of operation. For instance, urban and rural environments pose different sets of challenges to police work which should be reflected in the training of police officers (Crank, 1990; Huey and Ricciardelli, 2015). Second, societal or situational influences (e.g., occurrence of a terror attack, see Henry, 2002), changing policies (e.g., implementation of COVID-19 measures, see Laufs and Waseem, 2020; Frenkel et al., 2021), and technological advances (e.g., the integration of the body cam, see Koen et al., 2018; development of VR training, see Giessing, 2021) may call for specific adjustments in structure and content of police training. The concurrence of organizational, situational, and technological influences is what makes training practices unique to each law enforcement agency. As a consequence, the landscape of police training across law enforcement agencies is extremely diverse.

Although the diversity of training practices across European law enforcement agencies may explain why thus far no cross-cultural overviews of police training are collated, this does not mean that such an overview would be of no use. An overview featuring the commonalities and differences of European training practices highlights the diverse contexts in which law enforcement agencies educate and train police officers. This will, due to the variety in training practices, encompass a broad range of solutions law enforcement agencies have found for a broad range of issues with training. This may allow law enforcement agencies to learn from good practices in the training of other agencies and may help them to identify their own strengths and challenges in training more clearly. At the very least, law enforcement agencies may learn that they are not alone in particular aspects of training they struggle with and can be invited to join forces with other agencies to try and find improvements or solutions. These functions of a cross-organizational overview of training practices will be particularly salient if gained from the perspective of the actual law enforcement agencies and their personnel who conceptualize and deliver the training. In addition, the law enforcement agencies’ perspectives will benefit researchers on police training as it allows them to focus on current and practically relevant training areas that may necessitate further (scientific) attention. Evidence-based practice and practice-based evidence go hand in hand (or at least they should). As such, an overview of the intricacies of police training as experienced by law enforcement personnel such as training instructors is important and informative for police practitioners and researchers alike.

Manning (2009) eloquently argued that “police practices are well understood within the police world, and the reporting, designed for external audiences, is a shadowy figure” (p. 462). To shed light on the world of police training, we aim to gain insights into the commonalities and differences of European training practices and identify their strengths and challenges according to those who conceptualize, organize, and provide the training. Gaining insight into the strengths and challenges of police training as experienced by European law enforcement agencies will provide police practitioners and researchers an opportunity for optimizing the current state of police training.

## Materials and Methods

### Research Design

We utilized a qualitative research design to investigate the current state of police training at six European law enforcement agencies. We conducted individual interviews with police coordinators and instructors with the aim to identify strengths and challenges in police training.

Prior to conducting the interviews, we requested and received training-related material such as training and assessment manuals, lesson plans, training protocols, and training policies. We have studied these in detail to familiarize ourselves with the content and context in which police training takes place at each of the six law enforcement agencies. Reviewing the training-related material provided input for the interview guides and provided the interviewer with information on the language use and job-specific terminology expected from participants.

### Participants

In total, 21 semi-structured interviews were conducted on training sites at six European law enforcement agencies. The agencies were located in the Netherlands, Germany, Sweden, Romania, and Belgium. We interviewed a total of 23 participants (two female). 16 participants were police instructors with an average age of 39.75 years (*SD* = 6.59) and an average police work experience of 15.56 years (*SD* = 8.34). Seven were training coordinators (department heads, unit leaders, or instructors with conceptual tasks or other coordinative roles in police training) with an average age of 47.43 years (*SD* = 5.29) and an average police work experience of 26.86 years (*SD* = 7.32). The participants had knowledge of, and expertise in, the training of police cadets, the continued professional development of police officers, special forces officers, and police instructors. The profile of the participants of each law enforcement agency is further described in Table 1. To comply with confidentiality agreements with the law enforcement agencies, the participants and their respective organizations are anonymized. Ethical approval was obtained from the Social and Societal Ethics Committee of the Katholieke Universiteit Leuven as part of the SHOTPROS project (work package 9: ethics) which is funded by the European Union’s Horizon 2020 Research and Innovation Programme (Grant number: 833672).

**TABLE 1:** Profiles of the participants per law enforcement agency.

Organization	Participants
LEA 1	Instructor with conceptual training tasks (TC1).
	Instructors of continued professional development topics (I1, I2).
LEA 2	Instructor of shooting, close combat, and tactical training, with conceptual training tasks (TC2).
	Instructor of firearms instruction (I3).
	Instructor of tactical procedures of extreme violence and firearms instruction (I4).
	Instructor of firearms and equipment and fitness training (I5).
LEA 3	Weapon unit leader with coordinative training tasks (TC3).
	Instructor of firearms and equipment (I6).
LEA 4	Instructor coordinator with conceptual training tasks (TC4).
	Instructor and patrol officer (I7).
	Instructor of security detail personnel with organizational tasks (I8).
	Instructor of tactics, firearms instruction, first aid, and communication and border patrol officer (I9).
	Instructor of self-defense and tactical procedures and patrol officer (I10).
LEA 5	Head of instructor qualification unit for operational training (TC5).
	Instructor with conceptual tasks (TC6).
	Instructors of qualification and development of police instructors (I11, I12, I13, and I14).
LEA 6	Instructor with coordinative training tasks (TC7).
	Instructor of firearms and self-defense and military instructor (I15).
	Instructor of communications and military instructor (I16).

*“TC” refers to training coordinators; “I” refers to instructors. Coordinative training tasks refer to tasks in which training aspects are coordinated (e.g., scheduling, availability of instructors, personnel, location, etc.). Conceptual training tasks refer to tasks in which trainings are conceptualized (e.g., development of a training module, training plan, or training lesson, etc.).*

### Interview Guides

Based on the initial review of the training documents from law enforcement agencies, we developed two separate interview guides: one for interviews with police training coordinators with conceptual training tasks and one for interviews with police instructors. The interview guide for training coordinators consisted of discussion topics regarding the frequency, duration, and components of training, assessment and evaluation of officers, training for stressful situations and decision-making, an evaluation of the current training curriculum, and effective and innovative training practices. The interview guide for police instructors consisted of opinion inquiries regarding the overall experience as an instructor, their favorite parts of training, training methods they commonly implement in their training, and their views on what constitutes effective training. The interview guides can be requested from the first author.

### Procedure

To capture diverse perspectives of European police training, the first author visited the locations of six European law enforcement agencies in five different countries. To recruit participants for the interviews, we utilized purposive sampling where contact persons at each law enforcement agency referred us to key informants who further helped us recruit potential participants for the current study (Smith et al., 2009). All interviews were conducted by the first author, whose native language is German and who is fully proficient in English. To participate in the study, participants had to be (a) proficient in English or German, (b) provide operational police training as an instructor, or have a role in the conceptualization and organization of operational police training. Prior to visiting the location of the law enforcement agencies, our contact person ensured that at least one participant was in a position to be interviewed as training coordinator. Additional interviews with instructors were conducted based on the availability of instructors on location. We conducted all interviews face-to-face at the locations of the law enforcement agencies. Prior to conducting the interviews, we informed participants about the content and purpose of the study and asked for permission to audio-record the interview. All participants signed informed consent agreements prior to being interviewed. Due to time constraints of participants at one law enforcement agency, one of the interviews was conducted in a focus group setting with three participants (one training coordinator, two instructors) at the same time. For this interview, the interview guide for training coordinators was used.

### Analysis

We conducted an inductive thematic analysis following the steps described by Braun and Clarke (2006): familiarizing with the data, generating codes, constructing themes, reviewing potential themes, defining and naming themes, and producing the report. After transcription, translation of German interviews into English, and familiarization with the data, the transcripts were imported to ATLAS.ti 9 for analysis. The analysis started with open coding; we generated initial codes across the entire dataset. Initial descriptive codes were developed for any data segments that were meaningful to the researchers. In subsequent rounds of coding, we used our research questions to further specify the code labels. That is, we focused on data segments and code labels that referred to commonalities and differences of European training practices as well as signified strengths and challenges in these practices. Using thematic maps, provisional themes were explored in an iterative process to investigate the themes’ relationship between each other and to the research questions. Provisional themes that captured the dataset meaningfully and informed answers to the research questions were further employed as final themes. Next, we clearly named and defined the conceptualized themes and sub themes. To ensure that the final sub themes and their labels captured relevant aspects of the main themes, we wrote theme definitions summarizing the central idea (Terry et al., 2017). For validation purposes, a second researcher reviewed the transcripts and codes to ensure that results adequately reflected the original data.

## Results

The thematic analysis (Braun and Clarke, 2006; Terry et al., 2017) resulted in two main themes that were evident across all six law enforcement agencies: the organization of training and the delivery of training. In the following, the two main themes and their sub themes are presented separately. The main theme of organization of training relates to more formal, institutional information from the interviews regarding the structure and organization of training. The main theme of delivery of training reflects particular experiences and opinions of police coordinators and instructors about conducting and delivering the training. Table 2 provides an overview of the main themes, their sub themes, and the corresponding codes.

**TABLE 2:** Overview of main themes, sub themes, and their corresponding codes.

Main themes	Sub themes	Codes/Topics	N of total quotations	N of participants	N of agencies
Organization of training	Training curricula	Police academy training.	24	10	6
		Training frequency.	34	11	6
		Hierarchical organization structure.	21	11	5
		Curriculum development.	20	13	6
		Curriculum evaluation.	13	7	6
	Resource availability	Equipment availability.	12	8	6
		Instructor/personnel availability.	9	7	5
		Training facilities.	20	11	5
		Training time.	19	11	6
		Need to have officers on street.	6	4	3
	Training components	Practical skill components.	14	9	6
		Stress components.	19	11	6
		Decision-making components.	7	5	4
		Combined Training components.	19	10	6
		Dissatisfaction with components.	8	6	3
	Assessment	Assessment method.	15	6	6
		Assessment frequency.	8	5	4
		Assessment consequence.	15	8	6
Delivery of training	Role of the instructor	Perceived responsibility.	10	5	3
		Task description.	22	19	6
		Training preferences.	22	14	5
		Enjoyment.	22	13	5
	Didactical approaches and concepts	Linear pedagogy.	11	9	4
		Exploratory learning.	9	7	4
		Feedback.	17	10	5
		Repetition.	13	7	5
	Training environment	Importance of training environment.	10	8	4
		Tailored training environment.	11	5	4
		Realism in training environments.	10	8	5

*For each code, the table provides the number of total quotations, the number of participants (training coordinators and instructors) that the quotations came from, and the number of law enforcement agencies that these participants belonged to.*

### Organization of Police Training

#### Training Curricula

To understand the organization of formal training practices within policing, a specific look at the training curriculum of a law enforcement agency is key. A total of 33 quotations from the interviews were related to training curricula. The training curriculum outlines the components of training, as well as the frequency and duration that is spent on each of the training components. According to the interviewed law enforcement agencies, the education of a police officers is organized into two distinct phases: the basic formation of cadets at the police academy and the continued professional development of officers. At the law enforcement agencies that have been interviewed, the basic formation of cadets ranges from one to three and a half years depending on the requirements of the agency and consists of obtaining theoretical knowledge, practical knowledge, and internship-type on-duty experiences. Once graduated from the police academy, police officers continue to receive training on a yearly basis. During this continued professional development, the frequency and duration of the training is dependent on the law enforcement agencies requirements. For five of the interviewed agencies, the duration of training that patrol officers receive ranges from 16 to 48 h per year, organized into three to six training days per year. One law enforcement agency organizes their training on a weekly basis, where 2 h of training are carried out each week. A minimum of 6 h per month needs to be spent on practical training activities.

Although duration and frequency of the training differ across law enforcement agencies, similarities exist as well. All interviewed law enforcement agencies have a higher entity such as a (national) police board or (governmental) interior ministry that determines or approves training curricula. For instance, on a yearly basis the governing entity for police education provides a training focus that determines the contents that this particular law enforcement agency has to provide in their training for a particular year:

*“Every year we get [guidelines] from the national leader for practicing policy. And that’s tactics, firearms, and self-defense. And every year we get the information on what the year’s focus is going to be, for instance, terrorism, deadly violence or to increase the knowledge about psychological differences. So, we’ll get a document describing what kind of focus we should have during our practice for the year.” (TC4)*

When we asked training coordinators to reflect on their training curricula, the development thereof, and the delivery of amount and content of training to trainees, the responses across all six interviewed law enforcement agencies were remarkably similar. Training coordinators stated that the training curricula set the framework in which training delivery can take place. This framework consists of two factors; first, the frequency, duration, and components of the training curricula that are largely determined by the responsible external entities; secondly, the availability of resources to conduct the training prescribed in the training curricula. Training coordinators claim that what makes their training sufficient is not the development or state of the curriculum itself but what the instructors are able to achieve within the framework of the curriculum and with the resources available to them (see also training delivery):

*“I think with the time that we have here to train, we’re doing a good job, I think because everything depends. Everything is in the legislation about how many hours you can train a certain aspect of the formation and with the time that we have*. *If we had 2 years, it would be better, if we had a thousand rounds, it would be better.” (TC2)*

*“I think we’re doing well for what we can do at the moment. It can always be better; of that I am convinced. But we do not have the means to do it the way we want to.” (TC5)*

#### Resource Availability

With a total of 66 quotations, the availability of resources for training was a large topic for training coordinators and instructors: particularly the (limited) availability of instructors and personnel, training equipment, training facilities, training locations, and training time. For training coordinators and instructors, the shortcoming of these resources directly affects the quality of training, as well as the amount of knowledge and skills instructors are able to teach to cadets and police officers:

*“We have a short number of trainers, so that’s also something that is there. The quality of our training is under pressure.” (TC1)*

*“Due to the high number of students, it is not even possible to give every student a role-play on the respective topic. Unfortunately, that is just not doable, so [trainees] have to learn a lot by looking and watching. In terms of resources, this is unfortunately not possible any other way.” (TC3)*

*“If you come [to the training center] four times a year, then two of those times are tests, like the shooting test, the legal theory test. Then there’s not much time to learn something.” (I2)*

Training coordinators and instructors made clear wishes and suggestions on resources they require to improve the quality of their training:

*“My wish is to have a group of trainers only doing training because now I borrow the trainers from regular daily [work], you know, patrolling.” (TC4)*

*“The [instructors] need more logistics. We have no training infrastructures. We have no buildings, and we have no weapons.” (TC7)*

#### Training Components

To ensure that police officers continue to be well prepared for any on-duty incidents, law enforcement agencies provide training content that ensures that officers have the knowledge and skill to resolve situations they encounter on duty. The common training components discussed by all six law enforcement agencies include weapon handling, shooting, self-defense, arresting skills, tactical procedures (such as tactical movements during a building search), and communication. However, the way these components are trained differs across agencies. For instance, one law enforcement agency structures their formal yearly training content into five modules (one module for each training day), where three modules focus on the training of practical skills like weapon handling and shooting, equipment handling (e.g., multi-purpose baton, taser), and tactical procedures and movements. The other two modules consist of scenario-based training relating to the yearly training focus. In contrast, within the mandatory structure of spending a minimum of 6 h per month on practical skills (e.g., handcuffing, self-defense, use of force), another agency lets the unit leader of each police unit dictate the training components based on the needs of his or her officers.

According to training coordinators and instructors, using scenario-based training is seen as the most holistic and effective form of training. Scenario-based training is implemented in the delivery of training in each of the interviewed law enforcement agencies. Instructors make use of scenario training to combine training components:

*“The scenarios and role-play, everything of police training goes into it. So, you have two guns, you have knives, you have persons, cars and they have to act like they are on the street. And so that’s everything. When you are on the shooting range, you only shoot. When you are in the dojo, you only fight. But in the role plays, you do everything and it’s more complete.” (I4)*

The integration of stress inoculation training and decision-making is for most training coordinators and instructors an important part of these role-plays. Instructors use scenario trainings and role-plays to increase stress resilience of trainees and prepare them for stressful encounters on duty:

*“The trainees are prepared for [stressful situation] through specific scenario trainings in which [instructors] play with the stress. [Instructors] see how the trainees feel and control the stress. For those who can take it better, you go a little higher. The instructors can do that. You can actively increase [the stress] a bit or flatten it a bit, if you notice that they are not getting further. Through this targeted scenario training, [trainees] will become familiar with these stress levels.” (TC3)*

While performing under stress and making appropriate decisions in high-risk situations are integrated into the role-plays that instructors conduct, training coordinators stated that there is no set training component in the curricula which separately or specifically aims at preparing trainees for stressful situations on the job or teaches them appropriate decision-making and acting skills for on-duty encounters. To this end, most law enforcement agencies look to improve the current state of practice:

*“As I said, I think we’re at the beginning with decision-based training, we’re just getting started. So far, it has always been the case that we have provided a strict line and the solution to it was already predetermined. We are currently parting with [this approach] precisely because there is not always just one solution, there must be several solutions and several paths to reach these solutions.” (TC5)*

#### Assessment

To ensure that cadets and police officers maintain a sufficient standard of skill from the training components facilitated during the basic formation and the continued professional development, law enforcement agencies have various assessment measures in place. Across the law enforcement agencies that have been interviewed, these assessments entail written or theoretical tests of laws and regulations, shooting assessments on the shooting range, assessments of arresting and self-defense skills, the handling of certain gear such as the baton, physical fitness tests, and for some law enforcement agencies a combination of these.

Particularly during the continued professional development, not all European law enforcement agencies rely on formal assessment methods. One law enforcement agency mentioned that instead of having formal tests, police officers have evaluations with their unit leader on a yearly basis. The unit leader evaluates the officers’ on-duty performance and suggests areas in which additional training is needed. Based on this evaluation, the officer in question will be assigned to training courses that address his or her specific insufficiencies.

For law enforcement agencies that require formal assessment during the continued professional development, officers have to complete testing on a yearly basis. A particular assessment focus is placed on the weapon handling and shooting testing that takes place on a shooting range, which for one of the interviewed law enforcement agencies falls outside the range of yearly testing and instead takes place every 6 months. In case a police officer fails the shooting assessment, the officer has to hand in his or her service weapon and successfully repeat the shooting assessment before the weapon is returned and the officer is allowed to patrol the streets again.

However, due to resource limitation, such as the need for officers to patrol the streets, other areas of assessment do not have the same consequences as protocol dictates:

*“What happens if you fail the exam? In those areas, it is checked whether there’s a need for additional training. Often that is the case, and that would also be possible [to take on] for the instructors. But the authorities don’t send people because they have to be on the streets. Then that’s it, the person has deficits, but oh, maybe next year [it will be better].” (TC6)*

On the other hand, failing certain types of assessments may not have pre-dictated consequences at all:

*“The physical test is the only thing that hasn’t got consequences. You should get a positive [results], but if you don’t, no problem, you can still be on the street.” (TC1)*

### Delivery of Police Training

#### Role of the Instructor

According to the interviewed training coordinators and instructors, police instructors have an essential role in the conceptualization and delivery of a training session. To ensure that trainees learn effectively and efficiently, instructors have a wide range of demanding tasks to fulfill. Across the six interviewed law enforcement agencies, there are differences in tasks that instructors take on. For instance, two law enforcement agencies train their instructors to teach all components of training, whereas the other four law enforcement agencies have instructors that specialize and provide training solely in particular components such as shooting, self-defense, or tactical procedures. Similarly, some instructors specialize in the training of particular trainee groups like police recruits, regular police officers, or specialized teams such as special forces or undercover teams.

Independent of any specialization, all interviewed instructors feel a strong sense of responsibility associated with their role as a teacher. Instructors primarily felt that their responsibility in providing the training is to set up each training session with the aim to advance the knowledge and skill of the trainees and to create a safe environment in which trainees can learn:

*“The role of teachers is to make the trainings. You’re responsible for the safety in the training but also for making the good steps [in teaching]. […]. You have to think about what is the purpose of my training? And what do we want to reach at the end?” (I1)*

Next to feeling a strong sense of responsibility associated with their role as instructors, the instructors we interviewed seem to thoroughly enjoy providing training to trainees, resulting in 22 quotations related to enjoyment of their profession. The source of enjoyment that instructors share differs. For instance, three instructors explicitly stated that the training session itself is what they enjoy the most; particularly, having a training environment in which the interaction with the trainees is productive yet fun:

*“I think that it’s the most important for me as a teacher to have fun in my lessons, but also to [teach] them something and have a good interaction together.” (I1)*

Similarly, some instructors enjoy taking part in the learning process and witnessing the progress of their trainees the most. For yet others, the methodology of setting up a training is inherently enjoyable. For example, four instructors mentioned that the way they conceptualize the training is what brings them enjoyment in their profession as an instructor:

*“I like the way you build up a training. So that it’s useful and that it is as relevant as possible. That’s what I like. To play with that in your head before the training to get it so that it is all. So that it is a good training where people really learn. That’s what I enjoy most about giving training.” (I2)*

Although the source of enjoyment for their profession differs amongst instructors, the sense of responsibility to teach trainees relevant knowledge and skills prevails across all interviewed instructors and is reflected at the core of what instructors enjoy about their role as police instructors.

#### Didactical Approaches and Concepts

Because the delivery of training across and even within law enforcement agencies is as diverse as the frequency and duration specified in the training curricula across Europe, in the following, we provide an overview of the didactical approaches and concepts that European police instructors currently rely on in their training.

##### Linear Pedagogy

When setting up a training program or a single training session, the interviewed law enforcement agencies^1^ rely on a linear approach, building up a training in sequential stages from simple to complex. One instructor illustrated this approach using arresting techniques as an example:

*“So far, we’ve set up a lot of trainings from simple to complex. That means if you now want to learn an arrest technique that is a bit more complex, you have to start with the basics first, and then you build it up piece by piece. This means that the movements are sequenced. And at the end you do a learning objective check on this skill. You build up a bit of stress and see what stuck. The reality outside is that the possible suspect [you would use the arresting technique on] may move a little. That means, I won’t always be able to call on this great one technique that I have now trained for 2 h. I will have other stressors around it and maybe I will make other decisions as well.” (I14)*

While this approach can be considered common practice amongst the interviewed law enforcement agencies, some instructors and training coordinators see the difficulty in this approach. As is demonstrated in the quote above, teaching skills in a linear fashion to achieve the perfect technique is seldom realistic or applicable during on-duty incidents. To this end, one law enforcement agency explicitly mentioned that they aim to part with this approach which allows for more flexibility and realism in their training programs and sessions.

##### Exploratory Learning

Although the linear approach of teaching appears to be common practice amongst the interviewed law enforcement agencies, a few instructors approach their training sessions by placing exploration of movements rather than perfect technique at the center. Seven of the interviewed instructors (from four different law enforcement agencies) prefer to set up their training sessions in a way that lets their trainees explore different movements and solutions to problems themselves. One instructor describes his approach to exploration in training as follows:

*“I like to do it [this way]: I give a case and I’m not going to show you how to do it, you just do it. And then they do it. And it works out okay. But then I put in some input and say maybe like this and then they’ll try again and again. Not like provide [information on] this is the way that you’re always going to open a door, and this is the way you will always work an attack. If you do that, they are just going to copy me. And they might have good ideas too. So, I like to give them a chance to try. And if I see that it’s not good, I try to give them hints and then they find it out by themselves.” (I10)*

##### Feedback

With 17 quotations relating to feedback, instructors described that providing effective feedback is one of the most important tasks of the instructor during a training session. Giving feedback allows instructors to review their trainees’ performance, provide suggestions for improvement, and gather input from the trainees, while also ensuring that what is being learned by the trainees aligns with the purpose of the training session. Although most interviewed instructors mentioned that they utilize training time for giving feedback, the way they provide it differs from agency to agency. Some law enforcement agencies use unstructured feedback in which the role of the instructor is to debate the trainees performance and look for better solutions together, while other agencies prefer a more structured approach. For instance, one law enforcement agency has specific content that the instructors cover during a scenario-based training session:

*“We have a specific way of giving feedback and we are working on four points: And that’s safety, communication, movement, and treating the problem. And I think if you talk about those four aspects, you cover 90 percent of what you need to cover in a really small timeframe.” (TC2)*

Although the content of the feedback might be the same for instructors of the same law enforcement agency, the delivery of feedback oftentimes differs from instructor to instructor even within an agency. For instance, when using verbal feedback, four of the interviewed instructors explicitly mentioned that they prefer to have their trainees reflect on their performance themselves before the instructor provides input and recommendations. Four other instructors also noted the use of physical feedback as immensely important for their trainees to learn. For those instructors, physical feedback refers to feedback that trainees get from the training environment. To highlight this, one instructor (I2) provided the example of receiving a pain stimulus such as getting hit with non-lethal training ammunition when a trainee is not taking cover properly during a training scenario.

While the content, delivery, and type of feedback are important for instructors to consider, instructors have also mentioned that the timing of feedback is relevant in a training session. For instance, one instructor explained that he switches between providing feedback during a training scenario and providing feedback after the scenario is over:

*“Sometimes in some trainings, you will stop and say, OK, look how you’re standing, guys. Is that OK? Or what’s a better way to stand? And then you go, OK, you stand there and go further. So, you’re teaching in the moment. That’s a way of teaching and sometimes you need that […]. And sometimes it’s better to let it go and then afterward say, OK, look what you did. What more do you see? Is there someplace better you can stand? And then they [can] think about it.” (I1)*

##### Repetition

To ensure that trainees gain experiences in training and learn from situations in a safe, practical setting, instructors let trainees repeat a variety of training scenarios within a training session. For many of the interviewed training instructors (7), implementing repetition in the set-up of a training session is a staple part of their preparation. However, the opinions of instructors regarding the approach to repetition differ. For instance, one approach involves allowing trainees to repeat the same scenario to let them learn from their mistakes, while the other approach entails creating repetitions of slightly differing scenarios to provide new situations for trainees to solve. The former approach relates to a form of drill practice where trainees repeat the same task in the same setting until they are fully capable of solving the scenario. The approach of repeating the same scenario teaches trainees to apply a particular skill in a particular context. The latter approach — varying the environment and situation context of the scenario from one repetition to the next — allows trainees to explore solutions and make decisions regarding the use of the skill taught in the lesson.

In conclusion, because the delivery of training is not as heavily regulated as the components and skills that need to be trained, a lot of variation in the didactical and methodological approaches to training exists across and within the interviewed law enforcement agencies. Due to the limited regulation of training delivery, instructors have more freedom to use their expertise and experience to design and deliver a training session. Although common practices such as linear approaches to training exist in the training programs of law enforcement agencies, instructors are still able to use their expertise to set-up and deliver a training that aligns most with their perspective on how learning takes place effectively.

#### Training Environment

In preparation for a training session, the interviewed instructors described the importance of creating an effective training environment to enrich the quality of learning and motivation of trainees. To this end, instructors have differing opinions on what makes a training environment effective. For example, two instructors stated that an environment that allows trainees to make mistakes without judgment from peers or instructors is the most vital part. For another instructor, creating a positive environment in which trainees can have fun and are seen for their individual qualities is what makes the training environment effective for learning to take place:

*“I make very clear announcements. Definitely. But I also allow the individuality. I allow fun and joy because only in a positive learning atmosphere can you get the most out of people.” (I12)*

In addition, five instructors (from four different law enforcement agencies) mentioned that tailoring the training environment to the needs of the trainees is what allows for an environment to support a high quality of learning. To this end, one instructor described that the level of experience that the trainee groups have (e.g., recruits versus special forces) determines the way he sets up his training environment. The instructor explained that in a training session, he largely uses a variety of communication styles to adjust the environment to the level of the trainee groups. For instance, when training with special forces officers, his communication is more task-oriented and deviates little from the training objective. When training with recruits, he allows them the space to ask questions and reflect on problems and solutions.

While instructors aim to provide a supportive training environment through their guidance and communication, they also consider the physical training environment as a factor in creating an effective space for trainees to learn. This includes the setting in which the training takes place such as a shooting range or other training facilities but also the equipment available to conduct a training such as FX systems (a non-lethal combat training system that allows trainees to use shooting weapons with marking cartridges). To this end, instructors expressed concern regarding the level of realism they are able to achieve with the resources available to them:

*“For example, right now in the [training] centers that we have, we have different layouts, but they are still the same. And at one point, the person who is training is going to act like a robot. He knows the door is there. He knows he has two rooms, and I don’t know how many windows.” (I15)*

The familiarization with the fixed training facilities may lead trainees to dismiss important skills such as the careful scanning of a room that they would use in an unfamiliar, real-life setting. Similarly, when using FX systems, trainees are required to wear protection gear that they would not be wearing when they are patrolling the streets. Oftentimes this additional training equipment, though allowing for the safe use of shooting weapons in training, may hinder the level of realism of a training:

*“Whenever I have to disguise myself, because I put on protection helmets or something else, then you very often have the problem that the equipment interferes, it fogs up, I can no longer see anything. Also, I have no recognition of the hits because I am wearing thick clothes.” (I14)*

Instructors agree that although advances have been made, the issue of familiarization with fixed training facilities and the limited availability of current training equipment impedes the level of realism that instructors would like to achieve when designing training environments.

## Discussion

The organization and delivery of training practices described by training coordinators and police instructors of European law enforcement agencies reflect the diverse landscape of police training. This diversity is expressed in the training curricula, the organizational provisions of resources for training, and the didactical approaches to training delivery. In particular, the time recruits spend in basic training at the police academy highlights the diverse context of training across law enforcement agencies. While the amount of time recruits spend in the police academy is three and a half years for one law enforcement agency, police recruits at a different agency attend only for one year before assuming their position as patrol officers. This difference in education and training may be of particular concern for the increasing need of joint investigations in cross-border police cooperation (Meško, 2017). Cross-border investigations in which police officers of different agencies work together — relying on mutuality of knowledge of operational measures and investigative procedures — may prove challenging when knowledge and expertise of the officers differ tremendously across agencies.

While differences exist in police training across European law enforcement agencies, many of the current training practices share common principles. With the shared objective of providing training to develop the necessary proficiencies and improve the performance of police officers, law enforcement agencies across Europe focus on similar training components to ensure that officers are equipped with adequate knowledge and skills for duty. The interviewed agencies placed a particular focus on training of physical skills such as shooting, arresting, self-defense, and tactical procedures. The structuring of these skills into segmented training components (e.g., a training segment of the curriculum focusing only on learning to shoot, a different segment focusing only on learning self-defense skills) is a common principle in the organization of training. This finding is consistent with training practices of other European law enforcement agencies who also train particular components such as self-defense and arrest training, firearms training, and tactical training in isolated training segments (Staller et al., 2021). Similarly, the common principle in teaching of these components holds that training should be structured in a linear fashion moving from simple to complex, learning about the skill in a lecture-based setting, practicing the skill in a controlled setting such as the shooting range or the dojo, and then applying the skill in scenario-based role-plays (Renden et al., 2015). Taken together, the common principles law enforcement agencies share in the organization and delivery of training is the linear approach to learning.

The described overview of European police training practices represents the current state of training according to those who conceptualize and deliver it. Based on this current state of training, we discuss strengths and challenges of European police training with the aim to provide law enforcement agencies with examples of good practices, possible improvements, and solutions to challenges they may experience, as well as provide researchers with shortcomings in training that would benefit from further investigation.

### Strengths of Current Training Practices

Five of the six interviewed law enforcement agencies structure their training content of the continued professional development of police officers on the basis of a yearly training focus determined by an administrative police board for decisions on training curricula. Having a yearly content-specific training focus provides training coordinators and police instructors with current and realistic contexts in which they can structure and deliver the training of skills and procedures. Next to providing realistic and current training contexts, a yearly training focus determined by a national or regional advisory board is assumed to reflect the specific needs of each law enforcement agency’s region of operation, highlighting the necessity of training skills and procedures in the context of what has previously been an area of attention in that region. For instance, a multitude of police encounters with people with mental illnesses revealed that officers had difficulties recognizing, addressing, and interacting with people with mental illnesses (see Morabito, 2007; Livingston, 2016). A large number of these incidents may call a national or regional police board to decide to place a yearly training focus on interaction with people with mental illnesses — as has been the case for one of the interviewed law enforcement agencies. By maintaining an overview of the most pressing national or regional matters, administrative police boards can use the yearly training focus to shape their training content to be realistic, current, and relevant.

Another strength that the interviewed law enforcement agencies share is their ability to critically evaluate the efficacy of the current state of their training practices. To this end, training coordinators and instructors aim to identify shortcomings of their current practices and look to improve the state of training of their agencies. For instance, when recognizing that teaching police officers the perfect technique in a static setting did not ensure transfer to the complex and dynamic settings that officers encounter on the street (Pinder et al., 2015; Staller et al., 2021), the instructors began looking to adjust their approach to skill learning. Current changes that the interviewed law enforcement agencies aim to implement into their training include moving away from isolated technique mastery of a single skill (Abraham and Collins, 2011), implementing decision-based training (Helsen and Starkes, 1999; Johnsen et al., 2016), and changing pedagogical approaches from strictly linear to non-linear (see Koerner and Staller, 2018, 2021), combating the common principles that are still reflected in training curricula and delivery. The ability of training instructors, coordinators, and law enforcement agencies to be self-critical and identify areas of improvements in training facilitates the development of current practices.

Current police training and its delivery benefit from the diverse expertise of police instructors. Instructors’ perception of how learning takes place differs both across and within law enforcement agencies — similar to how one trainee’s learning preference differs from the next (Abraham and Collins, 2011). Because of their diverse perspectives on learning, each police instructor takes a unique approach to the delivery of their training. When law enforcement agencies provide their instructors with autonomy in the delivery of training, instructors have the opportunity to use their expertise and develop an approach that fits them best, rather than adhering to a prescribed teaching style that does not align with their expertise and preference. Giving police instructors the autonomy to use their expertise to guide a training session increases the chance that instructors are fully engaged in their practice and create motivating training environments for trainees (Klusmann et al., 2008; Christenson et al., 2012). This autonomy and space for diversity may indeed also be reflected in the high levels of enjoyment and motivation of instructors found in this study.

### Challenges of Current Training Practices

The overview of current training practices across European law enforcement agencies reveals numerous shared challenges that police coordinators and instructors face in the organization and delivery of police training. First, because the organization of police training — including the development and approval of training curricula — is governed by the hierarchical structure inherent to European law enforcement agencies, making modifications to the current state of training requires administrative effort (Martin et al., 2017; Shipton, 2019). For example, when instructors identify areas for improvement in their training, they have to take numerous steps to reach the level at which changes to training structures and curricula can be implemented. This process can be tedious and time-consuming, particularly if the deciding body for approval or rejection of modification to the training framework and curriculum is a governmental organization such as the internal ministry. Thus, although training coordinators and instructors may recognize room for improvement in their training practices and provide suggestions that align with developments and implementation recommendations in the scientific field (see Marenin, 2004; Koerner and Staller, 2021; Staller et al., 2021), for these suggestions to be reflected in training curricula and training delivery, numerous lengthy administrative hurdles have to be taken. Although the hierarchical structure of law enforcement agencies has the benefit of steering the content of training from an organizational position (e.g., by providing a yearly training focus), the drawback associated with this centralization is the slow and complex process for changes to take effect. While established organizational structures and processes are difficult to overcome or change — particularly in the world of policing (Chappell and Lanza-Kaduce, 2010) — law enforcement agencies may start by setting up internal “input groups” consisting of training coordinators and instructors to collect and monitor immediate challenges to the organization and delivery of training. The first function of such input group is to act as a direct link between the current state of training and the organizational entity in charge of training related decision-making. The second function of the input group is to differentiate challenges that require administrative structures and the attention of the organizational entity (e.g., changes to the training curriculum) and challenges to which solutions can be found and implemented on an immediate level [e.g., taking a learner-centered teaching approach in training sessions (Koerner and Staller, 2021)].

Another challenge that police instructors face points at the level of training delivery. When delivering a training, police instructors recognize the importance of providing realistic training to their trainees (Renden et al., 2015; Andersen et al., 2016). However, instructors are critical of the resources available to them to create realistic training environments (Cushion, 2018). For example, having to use the same training locations and facilities for scenario training allows trainees to familiarize themselves with the settings, providing a less effective low-stress and low-variance training environment (Adang, 2012; Staller and Zaiser, 2015; Staller et al., 2021). To this end, instructors wish for more flexibility in location and facilities in which they can conduct training. As availability of resources plays a part in the limited level of realism in training, an additional issue might be that instructors lack the knowledge or skill to make training sufficiently realistic with the resources available to them. By relying solely on physical aspects of the training environment, instructors dismiss other opportunities to make training realistic; for instance, designing training tasks that allow for motor, verbal, and cognitive skills to be trained in conjunction (Di Nota and Huhta, 2019). To address the issue of limited realism in training, law enforcement agencies should consider three possible solutions. First, although unlikely in the present economic climate (Cushion, 2018), law enforcement agencies could provide instructors with the resources they need to create realistic training (Di Nota and Huhta, 2019). Second, law enforcement agencies may look for cost-effective innovations such as the use of VR for realistic training (Giessing, 2021; Murtinger et al., 2021). Lastly, law enforcement agencies can strive to elevate the knowledge and skill of instructors to create realistic training with the (limited) resources available to them (see for instance Cushion, 2018 for an alternative approach to reality-based training).

The assessment and testing of knowledge and skills of police officers, particularly during the continued professional development, is another common challenge of current practices amongst law enforcement agencies. While law enforcement agencies put assessments into place to ensure that police officers possess a certain standard of skill, our findings showed discrepancies in the consequences for underperformance during those assessments. For instance, when failing the physical competency test, there are no consequences for officers of one law enforcement agency. Further, the assessments in place are rarely representative of the on-duty work that police officers perform (Lonsway, 2003; Tipton et al., 2013; Petersen et al., 2016; Koedijk et al., 2021). Similar to the modular, segmented structure of training components in the training curricula, currently applied assessment and testing methods evaluate isolated skills such as static shooting on the shooting range or self-defense technique evaluations. Law enforcement agencies can aim to improve their assessment practices by implementing common standards for underperformance and by reevaluating the design of their assessment methods to include representative testing environments (Staller et al., 2017; Koedijk et al., 2020).

## Conclusion

An overview of the current state of European police practices, their differences and commonalities, and strengths and challenges, depicts the landscape in which law enforcement agencies organize and deliver training. The findings emphasize the complexity of police training, including the common underlying principles that guide development and delivery of training, the administrative influence on developing and adjusting training curricula, and the wishes and needs of police coordinators and instructors for their training across law enforcement agencies.

European law enforcement agencies operate in diverse contexts and differ in the availability of resources to organize and deliver training. While the contexts and available resources differ, European law enforcement agencies face common challenges in training, such as having to undergo lengthy hierarchical, administrative processes to bring about changes to current training practices, achieving a limited level of realism in training, and having insufficient assessment standards. As each law enforcement agency has their distinct context in which they operate, a one-size-fits-all solution may not be helpful to overcome the challenges European law enforcement agencies share. To improve upon current training practices, generic recommendations, such as those provided in this paper (e.g., setting up a “input group” to influence administrative processes more concretely, providing additional training resources, alternative training systems, or trainer trainings to enhance the level of realism in training, and implementing common assessment standards and representative testing environments to enhance assessment practices), provide law enforcement agencies with initial directions for implementation of solutions. These generic recommendations can be adjusted by each law enforcement agency to encompass the wishes and needs, the distinct context of operation, and the available resources. Based on our findings on the strengths that European law enforcement agencies share, we are optimistic about improvements in police training. There is generally a solid structure by which training is organized and updated, law enforcement agencies possess self-critical and evaluative qualities at all levels involved with the daily practice of police training, and last, but certainly not least, police instructors are committed to optimizing the training environment for their trainees.

## Data Availability Statement

The datasets presented in this article are not readily available. Due to confidentiality agreements with participating law enforcement agencies and identifiable participant data in interview transcripts, the raw data obtained from the conducted interviews cannot be shared externally. Requests to access the interview guides should be directed to LK, l.kleygrewe@vu.nl.

## Ethics Statement

The studies involving human participants were reviewed and approved by the Social and Societal Ethics Committee of the Katholieke Universiteit Leuven as part of the SHOTPROS project. The patients/participants provided their written informed consent to participate in this study.

## Author Contributions

LK led the data collection and wrote the first draft of the manuscript. LK and RH performed the quantitative analysis. RH supervised the research process. RO, MK, and RH contributed to the manuscript revision. All authors contributed to the conception and design of the study and read and approved the submitted version.

## Conflict of Interest

The authors declare that the research was conducted in the absence of any commercial or financial relationships that could be construed as a potential conflict of interest.

## Publisher’s Note

All claims expressed in this article are solely those of the authors and do not necessarily represent those of their affiliated organizations, or those of the publisher, the editors and the reviewers. Any product that may be evaluated in this article, or claim that may be made by its manufacturer, is not guaranteed or endorsed by the publisher.

## Footnotes

1 Four law enforcement agencies explicitly described a linear approach in training, while the remaining two agencies did not explicitly discuss a linear approach to a training program or session during the interviews. Based on the reviewed training-related material from the law enforcement agencies themselves, these two agencies also use a linear approach in their training practices.

## References

[B1] Abraham A. Collins D. (2011). “Effective skill development: how should athletes’ skills be developed,” in *Performance Psychology: A Practitioner’s Guide*, eds Collins D. Button A. Richards H. (Oxford: Elsevier), 207–229.

[B2] Adang O. (2012). “Learning to deal with potentially dangerous situations: a situation-oriented approach,” in *Police Organization and Training*, eds Haberfeld M. Clarke C. Sheehan D. (New York, NY: Springer), 153–168.

[B3] Andersen J. P. Pitel M. Weerasinghe A. Papazoglou K. (2016). Highly realistic scenario-based training simulates the psychophysiology of real-world use of force encounters: implications for improved police officer performance. *J. Law Enforc.* 15 1–13.

[B4] Anderson G. S. Litzenberger R. Plecas D. (2002). Physical evidence of police officer stress. *Policing Int. J. Pol. Strat. Manag.* 25 399–420. 10.1108/13639510210429437

[B5] Birzer M. L. Tannehill R. (2001). A more effective training approach for contemporary policing. *Pol. Quar.* 4 233–252. 10.1037/amp0000186 29355356

[B6] Blumberg D. M. Schlosser M. D. Papazoglou K. Creighton S. Kaye C. C. (2019). New directions in police academy training: a call to action. *Int. J. Environ. Res. Public Health* 16 4941. 10.3390/ijerph16244941 31817578 PMC6950698

[B7] Braun V. Clarke V. (2006). Using thematic analysis in psychology. *Qual. Res. Psychol.* 3 77–101. 10.1191/1478088706qp063oa 32100154

[B8] Chappell A. T. (2008). Police academy training: comparing across curricula. *Policing Int. J. Pol. Strat. Manag.* 31 36–56. 10.1108/13639510810852567

[B9] Chappell A. T. Lanza-Kaduce L. (2010). Police academy socialization: understanding the lessons learned in a paramilitary-bureaucratic organization. *J. Contemp. Ethnogr.* 39 187–214. 10.1177/0891241609342230

[B10] Christenson S. Reschly A. L. Wylie C. (2012). *Handbook of Research on Student Engagement*, Vol. 840. New York, NY: Springer. 10.1007/978-1-4614-2018-7

[B11] Crank J. P. (1990). The influence of environmental and organizational factors on police style in urban and rural environments. *J. Res. Crime Delinq.* 27 166–189. 10.1177/0022427890027002004

[B12] Cushion C. J. (2018). Exploring the delivery of officer safety training: a case study. *Policing J. Pol. Pract.* 14 166–180. 10.1093/police/pax095

[B13] Di Nota P. M. Huhta J. M. (2019). Complex motor learning and police training: applied, cognitive, and clinical perspectives. *Front. Psychol.* 10 1797. 10.3389/fpsyg.2019.01797 31440184 PMC6692711

[B14] Frenkel M. O. Giessing L. Egger-Lampl S. Hutter V. Oudejans R. R. Kleygrewe L. (2021). The impact of the COVID-19 pandemic on European police officers: stress, demands, and coping resources. *J. Crim. Just.* 72 101756. 10.1016/j.jcrimjus.2020.101756 33100418 PMC7571900

[B15] Gershon R. R. Barocas B. Canton A. N. Li X. Vlahov D. (2009). Mental, physical, and behavioral outcomes associated with perceived work stress in police officers. *Crim. Just. Behav.* 36 275–289. 10.1177/0093854808330015

[B16] Giessing L. (2021). “The potential of virtual reality for police training under stress: a SWOT analysis,” in *Interventions, Training, and Technologies for Improved Police Well-Being and Performance*, eds Arble E. P. Arnetz B. B. (Hershey, PA: IGI Global), 102–124.

[B17] Helsen W. F. Starkes J. L. (1999). A new training approach to complex decision making for police officers in potentially dangerous interventions. *J. Crim. Just.* 27 395–410. 10.1016/s0047-2352(99)00012-4

[B18] Henry V. E. (2002). The need for a coordinated and strategic local police approach to terrorism: a practitioner’s perspective. *Pol. Pract. Res.* 3 319–336. 10.1080/1561426022000032088

[B19] Huey L. Ricciardelli R. (2015). ‘This isn’t what I signed up for’ When police officer role expectations conflict with the realities of general duty police work in remote communities. *Int. J. Pol. Sci. Manag.* 17 194–203. 10.1177/1461355715603590

[B20] Johnsen B. H. Espevik R. Saus E. R. Sanden S. Olsen O. K. (2016). Note on a training program for brief decision making for frontline police officers. *J. Pol. Crim. Psychol.* 31 182–188. 10.1007/s11896-015-9180-7

[B21] Klusmann U. Kunter M. Trautwein U. Lüdtke O. Baumert J. (2008). Teachers’ occupational well-being and quality of instruction: the important role of self-regulatory patterns. *J. Educ. Psychol.* 100 702. 10.1037/0022-0663.100.3.702

[B22] Koedijk M. Renden P. G. Oudejans R. R. Hutter R. I. (2019). Training for the job: evaluation of a self-defence training programme for correctional officers. *Ergonomics* 62 1585–1597. 10.1080/00140139.2019.1677947 31599188

[B23] Koedijk M. Renden P. G. Oudejans R. R. Kleygrewe L. Hutter R. I. (2021). Observational behavior assessment for psychological competencies in police officers: a proposed methodology for instrument development. *Front. Psychol.* 12 552. 10.3389/fpsyg.2021.589258 33732178 PMC7959728

[B24] Koedijk M. Stuurman H. F. Renden P. G. Hutter R. I. Strating M. Oudejans R. R. (2020). The physical competence test of the dutch national police: the effects of wearing a police uniform on test performance. *Pol. Pract. Res.* 21 264–278. 10.1080/15614263.2019.1658583

[B25] Koen M. C. Willis J. J. Mastrofski S. D. (2018). The effects of body-worn cameras on police organisation and practice: a theory-based analysis. *Pol. Soc.* 511 108.

[B26] Koerner S. Staller M. S. (2018). From system to pedagogy: towards a nonlinear pedagogy of self-defense training in the police and the civilian domain. *Secur. J.* 31 645–659. 10.1057/s41284-017-0122-1

[B27] Koerner S. Staller M. S. (2021). Police training revisited—meeting the demands of conflict training in police with an alternative pedagogical approach. *Pol. J. Policy Pract.* 15 927–938. 10.1093/police/paaa080

[B28] Laufs J. Waseem Z. (2020). Policing in pandemics: a systematic review and best practices for police response to COVID-19. *Int. J. Disas. Risk Reduct.* 2 101812. 10.1016/j.ijdrr.2020.101812 32839687 PMC7439012

[B29] Livingston J. D. (2016). Contact between police and people with mental disorders: a review of rates. *Psychiatr. Serv.* 67 850–857. 10.1176/appi.ps.201500312 27079990

[B30] Lonsway K. A. (2003). Tearing down the wall: problems with consistency, validity, and adverse impact of physical agility testing in police selection. *Pol. Quarter.* 6 237–277. 10.1177/1098611103254314

[B31] Manning P. K. (2009). Policing as self-audited practice. *Pol. Pract. Res. Int. J.* 10 451–464. 10.1080/15614260903378434

[B32] Marenin O. (2004). Police training for democracy. *Pol. Pract. Res.* 5 107–123. 10.1080/156142604200190261

[B33] Martin H. C. Rogers C. Samuel A. J. Rowling M. (2017). Serving from the top: police leadership for the twenty-first century. *Int. J. Emerg. Serv.* 6 209–219. 10.1108/IJES-04-2017-0023

[B34] McCoy M. R. (2006). Teaching style and the application of adult learning principles by police instructors. *Pol. Int. J. Pol. Strat. Manag.* 29 77–91. 10.1108/13639510610648494

[B35] Meško G. (2017). Police cooperation in the European Union, supported by strengthening the EU internal security’s external dimension. *Eur. J. Crime Crim. Law Crim. Justice* 25 109–121. 10.1163/15718174-25022108

[B36] Morabito M. S. (2007). Horizons of context: understanding the police decision to arrest people with mental illness. *Psychiatr. Serv.* 58 1582–1587. 10.1176/ps.2007.58.12.1582 18048560 PMC2811044

[B37] Murtinger M. Jaspaert E. Schrom-Feiertag H. Egger-Lampl S. (2021). CBRNe training in virtual environments: SWOT analysis & practical guidelines. *Int. J. Safety Secur. Eng.* 11 295–303. 10.18280/ijsse.110402

[B38] Ness J. J. (1991). The relevance of basic law enforcement training—Does the curriculum prepare recruits for police work: a survey study. *J. Crim. Just.* 19 181–193. 10.1016/0047-2352(91)90052-W

[B39] Nieuwenhuys A. Oudejans R. R. (2011). Training with anxiety: short-and long-term effects on police officers’ shooting behavior under pressure. *Cogn. Proces.* 12 277–288. 10.1007/s10339-011-0396-x 21431863 PMC3142543

[B40] Oudejans R. R. D. (2008). Reality-based practice under pressure improves handgun shooting performance of police officers. *Ergonomics* 51 261–273. 10.1080/00140130701577435 17896226

[B41] Paton D. (2009). *Traumatic Stress in Police Officers: A Career-Length Assessment From Recruitment to Retirement.* Springfield, IL: Charles C Thomas Publisher.

[B42] Petersen S. R. Anderson G. S. Tipton M. J. Docherty D. Graham T. E. Sharkey B. J. (2016). Towards best practice in physical and physiological employment standards. *Appl. Physiol. Nutr. Metab.* 41 S47–S62. 10.1139/apnm-2016-0003 27277567

[B43] Pinder R. A. Headrick J. Oudejans R. R. (2015). “Issues and challenges in developing representative tasks in sport,” in *Routledge Handbook of Sport Expertise*, eds Baker J. Farrow D. (Abingdon: Routledge), 269–281.

[B44] Renden P. G. Nieuwenhuys A. Savelsbergh G. J. Oudejans R. R. (2015). Dutch police officers’ preparation and performance of their arrest and self-defence skills: A questionnaire study. *Appl. Ergonom.* 49 8–17. 10.1016/j.apergo.2015.01.002 25766417

[B45] Shipton B. (2019). Police educators’ experiences of teaching: detailing differences between teacher- and learner-centred approaches. *J. Crim. Just. Educ.* 31 232–249. 10.1080/10511253.2019.1698755

[B46] Smith J. A. Flower P. Larkin M. (2009). Interpretative phenomenological analysis: theory, method and research. *Qual. Res. Psychol.* 6, 346–347. 10.1080/14780880903340091

[B47] Staller M. S. Zaiser B. (2015). Developing problem solvers: new perspectives on pedagogical practices in police use of force training. *J. Law Enforc.* 4 1–15.

[B48] Staller M. S. Koerner S. Heil V. Klemmer I. Abraham A. Poolton J. (2021). The Structure and delivery of police use of force training: a german case study. *Eur. J. Secur. Res.* 2 1–26. 10.1007/s41125-021-00073-5

[B49] Staller M. S. Zaiser B. Körner S. (2017). From realism to representativeness: Changing terminology to investigate effectiveness in self-defence. *Mart. Arts Stud.* 4 70–77. 10.18573/j.2017.10187

[B50] Terry G. Hayfield N. Clarke V. Braun V. (2017). Thematic analysis. *SAGE Handb. Qualit. Res. Psychol.* 2 17–37. 10.4135/9781526405555.n2

[B51] Tipton M. J. Milligan G. S. Reilly T. J. (2013). Physiological employment standards I. Occupational fitness standards: objectively subjective? *Eur. J. Appl. Physiol.* 113 2435–2446. 10.1007/s00421-012-2569-4 23263741

[B52] Waddington P. A. Badger D. Bull R. (2012). *The Violent Workplace.* Abingdon: Routledge. 10.4324/9781843926900

[B53] White M. D. Escobar G. (2008). Making good cops in the twenty-first century: emerging issues for the effective recruitment, selection and training of police in the United States and abroad. *Int. Rev. Law Comput. Technol.* 22 119–134. 10.1080/13600860801925045

[B54] Wilson J. M. Dalton E. Scheer C. Grammich C. A. (2010). *Police Recruitment and Retention for the New Millennium.* Santa Monica, CA: RAND Corporation. 10.7249/RB9546

